# Current trends in the treatment of hepatitis C: interventions to avoid adverse effects and increase effectiveness of anti-HCV drugs

**DOI:** 10.17179/excli2016-582

**Published:** 2016-10-14

**Authors:** Ammara Saleem, Muhammad Furqan Akhtar, Muhammed Fahd Mushtaq, Muhammad Saleem, Syed Taqi Muhammad, Bushra Akhtar, Ali Sharif, Sohaib Peerzada

**Affiliations:** 1 Faculty of Pharmaceutical Sciences, GC University, Faisalabad, Pakistan; 2 Faculty of Pharmacy, The University of Lahore, Lahore, Pakistan; 3 Institute of Pharmacy, Physiology and Pharmacology, University of Agriculture, Faisalabad, Pakistan

**Keywords:** hepatitis C, anti-HCV drugs, interferon, sofosbuvir, nanoparticles, pegylation

## Abstract

Viral hepatitis, an inflammatory liver disease, is caused by various genotypes of hepatitis C viruses (HCV). Hepatitis C slowly sprouts into fibrosis, which progresses to cirrhosis. Over a prolonged period of time compensated cirrhosis can advance to decompensated cirrhosis culminating in hepatic failure and death. Conventional treatment of HCV involves the administration of interferons. However, association of interferon with the adverse drug reactions led to the development of novel anti-HCV drugs given as monotherapy or in combination with the other drugs. Advances in drug delivery systems (DDS) improved the pharmacokinetic profile and stability of drugs, ameliorated tissue damages on extravasation and increased the targeting of affected sites. Liposomes and lipid based vehicles have been employed with polyethylene glycol (PEG) so as to stabilize the formulations as PEG drug complex. Sofosbuvir, a novel anti-HCV drug, is administered as monotherapy or in combination with daclatasvir, ledipasivir, protease inhibitors, ribavirin and interferon for the treatment of HCV genotypes 1, 2 and 3. These drug combinations are highly effective in eradicating the interferon resistance, recurrent HCV infection in liver transplant, concurrent HIV infection and preventing interferon related adverse effects. Further investigations to improve drug targeting and identification of new drug targets are highly warranted due to the rapid emergence of drug resistance in HCV.

## Introduction

Hepatitis C is an inflammatory disease of the liver that is often caused by various genotypes of HCV. Hepatitis B virus (HBV) and HCV infections affect 350 and 160 million people worldwide respectively. Infection with HCV and HBV are more common in low and middle income countries (Liaw and Chu, 2009[39]). Unavailability of the vaccine for HCV prophylaxis makes it harder to prevent the spread of infection in masses. The inability to reproduce HCV life cycle in-vitro and occurrence of HCV in different genotypes are the major reasons behind the failure to produce anti-HCV vaccine. However, treatment of HCV infection involves the administration of different drugs over a prolonged period of time (Lindenbach et al., 2005[40]). Major types of viral hepatitis, their causative agents, symptoms of exposure, incubation periods, transmission and diagnostic tests are depicted in Table 1(Tab. 1). 

Chronic hepatitis C is a potentially progressive disease. It is characterized by the gradual development of hepatic damage to fibrosis and progression to cirrhosis (Schuppan and Afdhal, 2008[56]). Compensated cirrhosis can progress to decompensated cirrhosis over a prolonged period, culminating into hepatic failure and death. Hepatocellular carcinoma (HCC) is the foremost cause of mortality in HCV infected patients (El-Serag, 2012[16]). HCC occurs predominantly in cirrhotic patients. However, several studies advocate the appearance of HCC in patients with bridging fibrosis without definite cirrhosis (Lok et al., 2009[42]).

Chronic HCV infection is a major cause of chronic liver disease and its associated deaths all over the world. Currently available highly active anti-HCV therapy eradicates the virus in 60 percent cases. Anti-HCV therapy also reduces the progression of hepatic damage to cirrhosis. There is an upsurge in mortalities worldwide due to the delayed detection, diagnosis and treatment of HCV infections. Transmission of HCV mainly occurs through the contact with blood and blood products in contrast to other hepatitis viruses (Alter and Klein, 2008[3]). The foremost cause of its transmission is the sharing of non-sterilized needles and syringes. However, transfusion-related hepatitis C has almost vanished with the advent of routine blood screening for HCV antibodies in 1991. Intravenous drug abuse is the most common risk factor now. Patients with high-risk sexual behavior are also at higher risk of HCV infection which may be due to its association with herpes simplex type-2 infection (Tohme and Holmberg, 2010[61]).

### Structure and life cycle of HCV

HCV is a spherical virus. It contains its genetic information in the form of a single stranded RNA (Henke et al., 2008[29]). Designing new drugs for the treatment of HCV infection requires understanding the life cycle of HCV (Scheel and Rice, 2013[55]). RNA is an easier to process form of genetic information as compared to DNA as there is no need for transcription. It reduces the need for specialized viral enzymes, which facilitate the transcription process. Other enzymes necessary for the synthesis of proteins are present in HCV. RNA of HCV is more prone to damage. It is advantageous to HCV as it allows facile mutations (Helle and Dubuisson, 2007[28]). This continuous alteration in RNA makes it harder for the human hosts to develop active or passive immunity against HCV (Scheel and Rice, 2013[55]). HCV contains a core in the form of RNA and a protein rich envelope which encloses the inner structure. Two envelope proteins, E1 and E2, allow the virus to attach to the liver cells (Bartosch et al., 2003[5]). E1 protein has certain regions which are easily mutated and therefore makes it harder for the immunity to develop. There are six major HCV genotypes, some of which are more common in certain regions and not frequently seen in other areas (Scheel and Rice, 2013[55]). Genotype 1 is the most common genotype worldwide and causes severe liver damage. HCV genotype 1 is also a major risk factor for hepatocellular carcinoma (Davidson et al., 1995[14]; Smith et al., 2014[58]; Messina et al., 2015[44]). Occurrence of various genotypes of HCV hinders not only the development of new vaccines but also affects the selection of alternative treatment strategies and may contribute to recurrent hepatic disease and post-transplant cirrhosis (Prieto et al., 1999[53]). Table 2(Tab. 2) shows different genotypes of HCV and their subtypes (Messina et al., 2015[44]). Important steps involved in the life cycle of HCV include attachment, entry and uncoating of the virus with the host cell membrane, translation and poly-protein processing, replication, assembly, egress and release of the viral RNA. The host cell cofactors which contribute to the HCV infection in tissue culture system include polypyrimidine tract binding protein 1 (PTBP1), Dead box RNA helicase (DDX3), Heat Shock Protein 70 (HSP70), Activating transcription factor 6 (ATF6), alpha Actinin 1 (ACTN1), Scavenger Receptor class B type I (SR-BI), Vesicle-associated membrane protein A and B (VAP-A/B), Microsomal transfer protein (MTP), Phosphoinositide-3-kinase (PI3K) (Georgel et al., 2010[21]).

### Pathophysiology of chronic hepatitis C

Chronic hepatitis C is not the consequence of direct destruction of hepatic cells by HCV. Rather, it results from an intermediate immune response that is large enough to encourage hepatic cellular injury. Such immune response is inadequate to eradicate the virus from its reservoir and culminates in fibrosis and hepatic cirrhosis. Quantitatively, HCV specific CD4 and CD8 T-cell responses are weaker in the chronic phase than the acute phase of infection. Patients with the poor response in acute phase are often asymptomatic (no jaundice) and are more likely to become chronic carriers than those exhibiting high immune response (Grebely et al., 2011[22]). HCV specific CD8 cells have an impaired effector function (both the secretion of antiviral cytokines and lytic activity). The effectiveness of a combination of interferon and ribavirin is probably explained by its antiviral activity and restoration of HCV specific immune response. 

### Therapeutic targets for treatment of HCV infection

Entry of viral RNA in the liver cells can be blocked through entry inhibitors which include neutralizing anti-receptor antibodies and receptor antagonists (Iwamoto et al., 2014[32]). Immune modulators include interferon derivatives, TLR agonists, cytokines, and therapeutic vaccines (Yang et al., 2011[66]). Helicase inhibitors block the subgenomic replication of virus (Najda-Bernatowicz et al., 2010[47]). RNA interference is a technique in which small RNAs are used to alter the gene expression for the treatment of chronic hepatitis C and include drugs such as siRNA antisense oligonucleotides and miRNA 122 antagonists (Castanotto and Rossi, 2009[10]). Anti-HIV drugs such as protease inhibitors have also shown high potential to treat chronic hepatitis C (D'Avolio et al., 2013[13]). Sofosbuvir, a polymerase inhibitor, is a highly effective anti-HCV drug (Pawlotsky, 2014[50]). Cyclophilin inhibitors suppress the replication of HCV and include cyclosporin A (Chatterji et al., 2015[11]). Potential treatment of chronic hepatitis C may also involve drugs which target glycosylation through inhibition of alpha-glucosidase (Dwek et al., 2002[15]). Ribavirin, a guanosine analogue, is a prodrug and it blocks RNA synthesis (Hoofnagle, 2002[31]).

## Pharmaceutical Interventions for the Improvement of Anti-HCV Therapy

Advances in drug delivery systems (DDS) significantly improved the pharmaceutical issues associated with the use of antiviral therapy related conventional DDS. Advanced DDS involve particulate carriers comprised of the lipid polymers. Advanced DDS improved the pharmacokinetic profile and stability of drugs, tissue damages on extravasation and drug targeting of the affect sites (Table 3(Tab. 3); References in Table 3: Liu et al., 2006[41]; Harris and Chess, 2003[26]; Veronese and Pasut, 2005[62]; Milla et al., 2012[45]; Guo et al., 2015[25]; Sánchez et al., 2003[54]; Sheffield, 2001[57]; Hashim et al., 2010[27]; Gaillard et al., 2014[18]; Gunaseelan et al., 2010[24]). Advanced DDS comprising liposomes and lipid based formulations are designed in such a way that more stabilized PEG drug complex is formed by linking PEG with interferons (Allen and Cullis, 2004[1]).

Classical liposomes also known as 1^st^ generation liposomes exhibit several limitations related to the variability in pharmacokinetics of anti-HCV drugs. Serum proteins affect the drug entrapment in classical liposomes. Incorporation of cholesterol resolved this problem in such a way that it entrapped the drug contents and reduced the leakage of drug. The rapid clearance of drugs from 1^st^ generation liposomes was also problematic. Uptake of these liposomes by mononuclear phagocytes in the liver and spleen results in exacerbation of site specific toxicity and decrease in drug distribution to other tissues. Increasing the half-life of circulating liposomes resolved this issue as mononuclear phagocytes were blocked (Kumari et al., 2010[36]). Triggered release of the liposomal DDS allows anti-HCV drugs to reach target sites and improves therapeutic outcomes. There are two types of triggers for the release of drug from liposomal DDS (Allen and Cullis, 2013[2]). Heat, ultrasound and light may act as remote triggers for the release of liposomal DDS, whereas local triggers include enzymes and pH changes. 

Development of nanoparticles improves drug targeting, increases efficacy and prevents the adverse effects of anti-HCV drugs and the drugs used against HCV induced cancer. Nanoparticles are taken up by the liver, spleen and reticuloendothelial system. The particles having diameters 100 nm or less are highly accessible to the targeted site. Nanoparticles exhibiting hydrophobicity are taken up by the liver, whereas those showing lipophilicity are taken up by the spleen and reticuloendothelial system (Zhou et al., 2011[68]). Drugs used against HCV induced cancer are designed so as to increase the permeability and retention at the target site, inhibiting angiogenesis and targeting tumor vasculature (Brannon-Peppas and Blanchette, 2012[7]).

FDA has approved two recombinant alpha interferons as initial therapy for chronic hepatitis C. It was recommended in 1991 that three million units (MU) of interferon alfa-2b could be given three times per week for a period of 6 months (McHutchison et al., 2009[43]). The use of 3 MU of interferon alpha-2a was also approved in 1996, for subcutaneous administration three times per week for a period of 12 months. Other drugs investigated for the treatment of HCV infection included interferon alpha-n1, consensus interferon, leukocyte-derived interferon and several beta interferons. However, the standardization and evaluation of comparative biological potency of various interferons remain problematic.

## Pharmacological Interventions for the Improvement of Anti-HCV Therapy

A combination of interferon and ribavirin has been used extensively against HCV infections of genotype 2 and 3 which is highly correlated with the antiviral response in most patients. However, interferon therapy is associated with severe adverse reactions such as fatigue, anxiety, depression, hair loss and thrombocytopenia. Moreover, interferon is contraindicated in patients with hepatic decomposition, auto-immune diseases and some psychiatric illnesses (Bull et al., 2009[8]; Yamane et al., 2008[65]). These factors prompted the investigation for novel anti-HCV drugs.

### Sofosbuvir 

Sofosbuvir is an oral, direct acting nucleotide analogue inhibitor of the nonstructural HCV protein NS5B polymerase (Pawlotsky, 2013[51]; Herbst and Reddy, 2013[30]). Sofosbuvir was investigated as a substitute for interferon. Sofosbuvir was concurrently administered with ribavirin for the assessment of safety and efficacy in patient with HCV genotype 2 and 3. It was found that sofosbuvir prevented the recurrence of HCV infection when used in combination with ribavirin (Curry et al., 2015[12]). The dose of sofosbuvir used in HCV infection is 400 mg, whereas the dose of ribavirin depends upon the body weight. Ribavirin is administered as 1000 mg per day when the body weight is less than 75 kg. The dose of ribavirin is increased to 1200 mg per day for body weight of 75 kg or more. Sofosbuvir and ribavirin are highly effective in HCV genotype 2. However, this combination is less effective in HCV genotype 3 and the patients with liver cirrhosis than HCV genotype 2 (Osinusi et al., 2013[49]). 

Sofosbuvir and ribavirin combination therapy is more effective and safe alternative because it reduces the level of circulating RNA beyond the quantification limit. Sofosbuvir associated adverse reactions include insomnia, fatigue, rashes and anemia. These adverse reactions were similar in patient with or without liver cirrhosis (Lawitz et al., 2014[38]; Jacobson et al., 2013[33]). Sofosbuvir is also very effective in patients with liver transplant suffering from recurrent HCV infections (Berenguer, 2015[6]).

### Combination of sofosbuvir and ribavirin

Various studies demonstrate that a combination of sofosbuvir and ribavirin is the most effective treatment of HCV infection (Zeuzem et al., 2014[67]; Gane et al., 2013[19]). An important investigation involved the previously treated and untreated patient with HCV genotype 1, 2 and 3. The patients with HCV genotype 2 and 3 infection treated with sofosbuvir and ribavirin exhibited undetectable levels of viral RNA. The patients receiving the pegylated interferon along with sofosbuvir and ribavirin had sustained virologic response. The inclusion of interferon in therapy had no significant effect on the virologic response. Sofosbuvir monotherapy exhibited a detectable level of the viral RNA in patients with genotype 2 and 3. The monotherapy with sofosbuvir also reduced the level of the hemoglobin (Neri et al., 2010[48]; Lawitz et al., 2013[37]). A combination of sofosbuvir and ribavirin inflicted urethral injury in previously untreated patients with HCV genotype 1 (Gane et al., 2013[19]). Patients with HCV genotype 3 infections demonstrated the symptoms of angina pectoris after the treatment with sofosbuvir, ribavirin and interferon (Kattakuzhy et al., 2015[34]).

### Combination of sofosbuvir and ledipasivir 

Interferons cause various adverse reactions such as hemolytic anemia, fatigue, pruritus and rashes. Exclusion of interferons and ribavirin from HCV therapy is expected to reduce these adverse effects. Ledipasivir inhibits nonstructural protein NS5A while sofosbuvir inhibits a nonstructural protein NS5B present in HCV (Gane et al., 2014[20]). Ledipasivir is highly active against HCV genotype 1a and 1b, whereas sofosbuvir is active against genotype 1 (Sulkowski et al., 2014[60]). The combination of ledipasivir and sofosbuvir with or without ribavirin exhibited highly sustained virologic response among the patients with HCV genotype 1. A combination of ledipasivir and sofosbuvir was found to be more effective in patients with HCV genotype 1 than a combination of ledipasivir, sofosbuvir and ribavirin. The patients treated with ledipasivir and sofosbuvir exhibit some common adverse reactions such as fatigue, headache, insomnia and nausea however, patients treated with ledipasivir, sofosbuvir and ribavirin showed an upsurge of adverse effects such as fatigue, asthenia, cough, anemia and rashes (Zeuzem et al., 2014[67]).

### Combination of sofosbuvir and daclatasvir

All oral combination therapy is desirable for patients with chronic HCV infection (Poordad et al., 2013[52]). Daclatasvir is an HCV NS5A replication complex inhibitor (Guedj et al., 2013[23]). A combination of daclatasvir and sofosbuvir with or without ribavirin was investigated in patients infected with HCV genotype 1, 2, or 3. Both the drugs are the potent anti-HCV drugs and have broad genotypic coverage. Daclatasvir and sofosbuvir exhibited a sustained antiviral response in patients with HCV genotype 1a, 1b and 3 who had shown poor response to previous treatment with telaprevir and boceprevir. The most common side effects of this combination include the fatigue, nausea, gastroenteritis, colitis and stroke.

### Combination of ribavirin with ritonavir, ombitasvir and dasabuvir 

Ombitasvir is an inhibitor of the NS5A. Ritonavir is a protease inhibitor and a potent inhibitor of CYP-450 so that it augments the response of other drugs (Kempf et al., 2007[35]). Combination therapy of ritonavir with ombitasvir, dasabuvir and ribavirin has been investigated in previously treated and treatment naive patients of HCV genotype 1 infection and HCV infected cirrhotic patients. It was found that there was a high virologic response to treatment in the HCV genotype 1 patients. Minor adverse effects appeared in those patients and did not allow any discontinuation of therapy. There was also no drug resistance (Andreone et al., 2014[4]). The adverse effects occurring in those patients included nausea, insomnia, purities and asthenia. This favorable adverse effect profile advocates the oral use of protease inhibitors with or without interferon and ribavirin. It is also found that the multi-targeted regimens were highly effective in patient with HCV genotype 1 exhibiting no cirrhosis.

### Combination of sofosbuvir and daclatasvir therapy for liver transplant

Hepatitis is a leading cause of liver transplant (Wong et al., 2015[63]). Pegylated interferon and ribavirin has been used in liver transplant recipients. However, severe adverse effects and low antiviral activity are the major limitations of treatment with pegylated interferon and ribavirin (Xirouchakis et al., 2008[64]). Protease inhibitors, such as boceprevir and telaprevir have been investigated for recurrent HCV in liver transplant. These drugs had a moderate antiviral response when given in combination with interferon and ribavirin. However, use of strong inhibitors of CYP3A4 such as protease inhibitors resulted in serious drug-drug interactions. The study demonstrated that a regimen comprising sofosbuvir and daclatasvir was highly effective in liver transplant patients. Safety and efficacy profile of this regimen makes it the most appropriate treatment option for liver transplant patient with recurrent HCV infection (Fontona et al., 2013[17]).

### Combination of sofosbuvir and daclatasvir for HCV in patients co-infected with HIV

Pegylated interferon and ribavirin may not exhibit high virologic response in HCV patients co-infected with HIV (Carrat et al., 2004[9]). A combination of daclatasvir and sofosbuvir was investigated in these patients. Daclatasvir, an inhibitor of the NS5A and sofosbuvir, an inhibitor of NS5B, were given in combination to attain high virologic response in patients with HCV genotype 1, 2 and 3. The investigation showed that daclatasvir and sofosbuvir had a highly sustained virologic response in HCV patients co-infected with HIV-1 genotypes 1, 2, 3 and 4. This combination had a lower virologic response in patients with HCV genotype 3 with cirrhosis than the non-cirrhotic patients. The common side effects associated this regimen were fatigue, headache and nausea (Smith et al., 2015[59]).

### Combination of sofosbuvir and ribavirin for HCV in patients co-infected with HIV

Interferon based regimens are still an important choice in patients with HCV genotypes 1 or 4 co-infected with HIV. Interferon-free regimens approved for patients with HCV genotype 2 or 3 co-infected with HIV include a combination of sofosbuvir and ribavirin. Severe toxic effects and drug interactions with anti-retroviral therapy limit the clinical usefulness of these regimens. An oral combination of sofosbuvir and ribavirin were investigated in patients with HCV co-infected with HIV. Sofosbuvir and ribavirin achieved highly sustained virological response after 12 weeks of treatment. These findings suggest that administration of sofosbuvir and ribavirin for 12 or 24 weeks is highly effective in chronic hepatitis C patients co-infected with HIV genotypes 1-4 (Molina et al., 2015[46]).

## Conclusion

It can be concluded that the use of interferon in the treatment of chronic hepatitis C exhibits a large number of adverse effects. Pegylation of interferon increases the efficacy and ameliorates the adverse effects of interferons. Interferon can be replaced with nonstructural polymerase NS5A and NS5B inhibitors such as sofosbuvir and daclatasvir respectively. Sofosbuvir used as monotherapy or in combination with ribavirin, daclatasvir and ledipasivir is highly effective in interferon resistance, recurrent HCV infections in liver transplant and preventing interferon related adverse effects. Emergence of resistance to existing anti-HCV therapy and their adverse effects warrant further investigations in dosage form development. It is necessary to increase the efficacy of anti-HCV drugs through active or passive targeting and the development of novel drugs to remediate this global calamity.

## Notes

Ammara Saleem and Muhammad Furqan Akhtar (Faculty of Pharmacy, The University of Lahore, Off Defense Road, Lahore, Pakistan; e-mail: furqan.pharmacist@gmail.com) contributed equally as corresponding authors.

## Conflict of interest

Authors declare that they have no conflict of interest.

## Figures and Tables

**Table 1:**
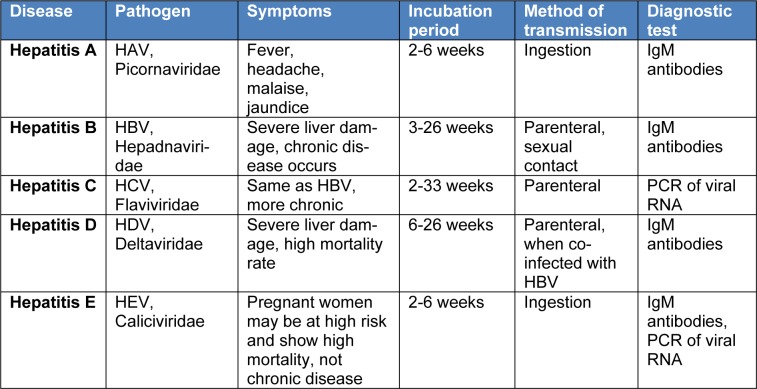
Features of different types of viral hepatitis

**Table 2:**
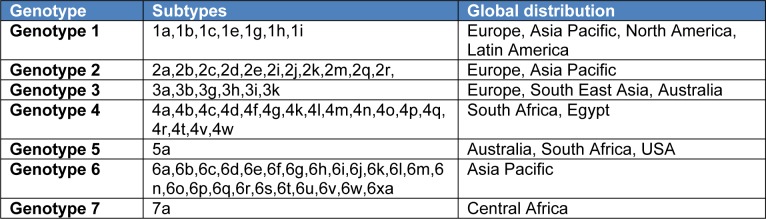
Genotypes of HCV with different subtypes and their global distribution

**Table 3:**
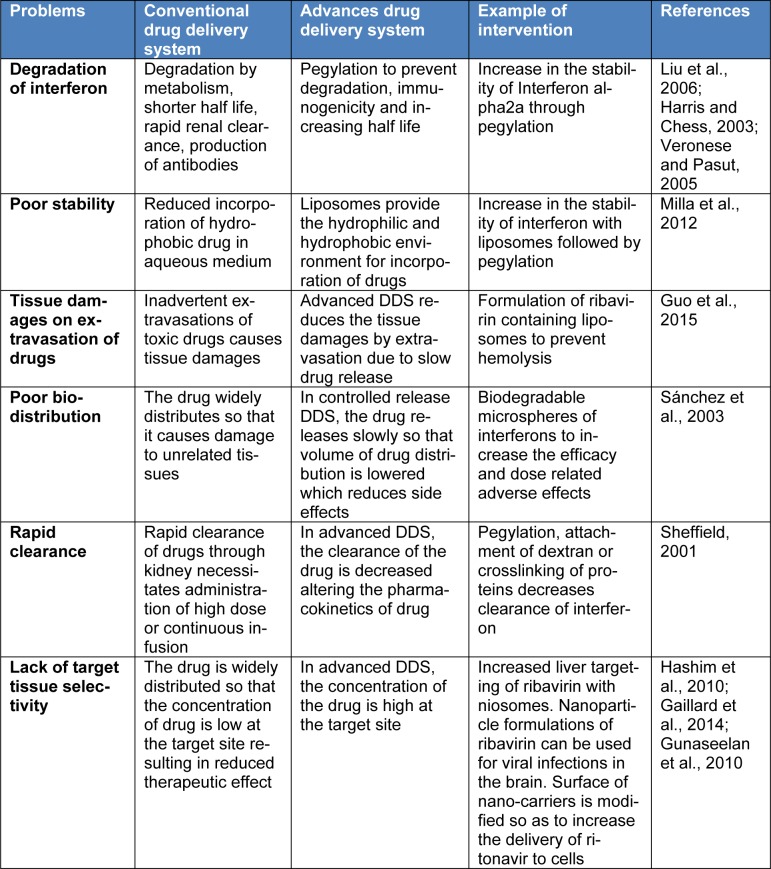
Pharmaceutical interventions for improving safety and efficacy of anti-HCV drugs
